# Poor efficacy response to trastuzumab therapy in advanced gastric cancer with homogeneous HER2 positive and non-intestinal type

**DOI:** 10.18632/oncotarget.16567

**Published:** 2017-03-25

**Authors:** Chen Xu, Yalan Liu, Dongxian Jiang, Qian Li, Xiaowen Ge, Ying Zhang, Jie Huang, Jieakesu Su, Yuan Ji, Jun Hou, Shaohua Lu, Yingyong Hou, Tianshu Liu

**Affiliations:** ^1^ Department of Pathology, Zhongshan Hospital, Fudan University, Shanghai, China; ^2^ Department of Pathology, School of Basic Medical Sciences and Zhongshan Hospital, Fudan University, Shanghai, China; ^3^ Department of Oncology, Zhongshan Hospital, Fudan University, Shanghai, China

**Keywords:** gastric cancer, HER2, heterogeneity, trastuzumab, efficacy

## Abstract

**Introduction:**

Factors affecting trastuzumab efficacy in advanced gastric cancer (GC) are largely unknown. Heterogeneity is a notable feature of HER2 in GC. Whether the heterogeneity influences trastuzumab efficacy is still unknown.

**Results:**

The HER2^homogeneous^ group and HER2^heterogeneous^ group showed no statistical difference in RR (46.4% vs 55.0%, *P* = 0.558), PFS (5.80 vs 6.30 months, *P* = 0.804) and OS (16.00 vs 16.00 months, *P* = 0.787). The Lauren^intestinal^ group and Lauren^non-intestinal^ group demonstrated no discrepancy in PFS (6.00 vs 6.00 months, *P* = 0.912) and OS (16.50 vs 14.00 months, *P* = 0.227). However, by combining HER2 heterogeneity and Lauren classification, PFS and OS of HER2^homogeneous^/Lauren^non-intestinal^ subgroup was the shortest among the 4 subgroups (*P* = 0.012 and *P* = 0.037), which was much shorter than the other patients (PFS:3.00 vs 6.30 months, *P* = 0.003; OS: 4.50 vs 16.50 months, *P* = 0.004). Univariate and multivariate analysis showed that HER2 heterogeneity combined with Lauren classification was an independent prognostic factor in both PFS (*P* = 0.031 and *P* = 0.002) and OS (*P* = 0.039 and *P* = 0.013).

**Materials and Methods:**

48 patients with HER2 positive advanced GCs accepting trastuzumab treatment were retrospectively analyzed. Based on HER2 heterogeneity, the patients were divided into a HER2^homogeneous^ group and a HER2^heterogeneous^ group. Response rate (RR), progression free survival (PFS), and overall survival (OS) were compared. Main clinicopathological factors including Lauren classification were subjected to subgroup analysis.

**Conclusions:**

HER2 heterogeneity alone may not correlate with trastuzumab efficacy in HER2 positive advanced GCs. HER2 heterogeneity combined with Lauren classification may help to identify a subgroup with poor response to trastuzumab which is homogeneous HER2 positive and non-intestinal type.

## INTRODUCTION

Gastric cancer (GC) is one of the most lethal cancers worldwide [1]. As the second and third most common cancer among male and female respectively, it is the second leading cause of cancer death for both genders in China [2].

Many GC patients in China present with inoperable or metastatic tumors during their first visit. For them, the prognosis is dismal with a median overall survival (OS) of less than 12 months [3]. Recently, trastuzumab was established as a new standard care for the first line treatment of patients with advanced GCs [4]. The targeted therapy has been proved to significantly improve the survival of patients with HER2 positive GCs [5].

HER2 status has been studied extensively in GC, and criteria for HER2 interpretation has been established and standardized during these years [6–9]. HER2 is associated with Lauren classification, and HER2 positive GCs are mainly intestinal type [10–12]. Additionally, heterogeneity is more common in GC than in breast cancer, which was estimated to be from 30% to up to 79.3% of HER2 positive GCs [13–15].

As a targeted regimen, trastuzumab provides modest overall survival benefit for HER2 positive advanced GCs [16]. Factors to affect the efficacy are largely unrevealed. HER2 heterogeneity has not been well studied as a potential factor in trastuzumab response in GC. In the present study, we investigated the impact of HER2 heterogeneity as well as other vital clinicopathological factors including Lauren classification on trastuzumab responses and clinical outcomes in patients with HER2 positive advanced GCs. We hope to find possible predictive factors for trastuzumab efficacy in GC.

## RESULTS

### Patient characteristics

From February 2010 to March 2016, 85 patients with advanced GC received trastuzumab treatment. Pathological specimens of 57 patients were available. Among them, 9 patients were out of follow up. Finally, there were 48 eligible patients. 36 were male. The age ranged from 40 to 87 (median: 64.0, mean: 63.0). Among them, 39 were intestinal type, while the left 9 were non-intestinal type (7 mixed type and 2 diffuse type). All the patients received trastuzumab as the first line treatment. Trastuzumab was used within the first line of treatment in 27 patients. The remaining 21 patients received continuation of trastuzumab beyond the first line progression (second line). RR of total patients was 50.0%. The median PFS and OS was 6.00 months and 16.00 months respectively. Patient characteristics including demographics, pathological features, and treatment profiles were shown in Table 1.

**Table 1:** Clinicopathological features of the patients

	Total	HER2^homogeneous^	HER2^heterogeneous^	*P* value
Gender				0.311
Male	36 (75.0%)	19 (67.9%)	17 (85.0%)	
Female	12 (25.0%)	9 (32.1%)	3 (15.0%)	
Age				0.979
< 60	16 (33.3%)	9 (32.1%)	7 (35.0%)	
≥ 60	32 (66.7%)	19 (67.9%)	13 (65.0%)	
Tumor location				0.507
GEJ	17 (35.4%)	11 (39.3%)	6 (30.0%)	
Other stomach	31 (64.6%)	17 (60.7%)	14 (70.0%)	
Lauren				0.348
Intestinal	39 (81.3%)	24 (85.7%)	15 (75.0%)	
Non-intestinal	9 (18.7%)	4 (14.3%)	5 (25.0%)	
Differentiation				0.269
Moderate	33 (68.8%)	21 (75.0%)	12 (60.0%)	
Poorly	15 (31.2%)	7 (25.0%)	8 (40.0%)	
HER2 status				0.636
IHC 3+	43 (89.6%)	26 (92.9%)	17 (85.0%)	
IHC 2+/FISH+	5 (10.4%)	2 (7.1%)	3 (15.0%)	
Radical resection				0.499
Yes	12 (25.0%)	6 (21.4%)	6 (30.0%)	
No	36 (75.0%)	22 (78.6%)	14 (70.0%)	
Number of metastatis				0.430
< 3	33 (68.8%)	18 (64.3%)	15 (75.0%)	
≥ 3	15 (31.2%)	10 (35.7%)	5 (25.0%)	
Response				0.558
CR/PR	24 (50.0%)	13 (46.4%)	11 (55.0%)	
SD/PD	24 (50.0%)	15 (53.6%)	9 (45.0%)	
Trastuzumab administration				0.302
First line	27 (56.3%)	14 (50.0%)	13 (65.0%)	
First and second line	21 (43.7%)	14 (50.0%)	7 (35.0%)	
Median cycles of trastuzumab therapy (median, range)	7.0 (1–34)	7.0 (1–34)	9.0 (2–20)	0.429
Median cycles of chemotherapy(median, range)	7.0 (1–34)	6.5(1–34)	8.0 (2–20)	0.490
mPFS (95%CI,months)	6.00 (5.46–6.55)	5.80 (3.87–7.73)	6.30 (1.92–10.68)	0.061
mOS (95%CI,months)	16.00 (13.68–18.32)	16.00 (8.74–23.26)	16.00 (13.59–18.41)	0.787

Abbreviations: GEJ: gastric esophagus junction; CR: complete response; PR: partial response; SD: stable disease; PD: progressive disease; mPFS: median progression free survival; mOS: median overall survival.

### HER2 status

Based on HER2 status, the 48 patients were divided into two groups: a HER2^homogenous^ group (*n* = 28) and a HER2^heterogeneous^ group (*n* = 20). Clinicopathologic characteristics of the two groups were generally balanced (Table 1).

The distribution of patients depending on HER2 status and sample type was shown in Table 2. Briefly, in the 48 patients, 43 were IHC 3+, and the left 5 were IHC 2+/FISH+. Among the IHC 3+ patients, 33 were only with biopsy specimens because of unresectable advanced/metastatic lesions during their first visit (20 HER2^homogeneous^ and 13 HER2^heterogeneous^). The left 10 patients received radical resection before recurrence/metastasis, and resected specimens were available. Among them, 6 exhibited homogeneous HER2 expression, and 4 was HER2 heterogeneous.

**Table 2:** Distribution of patients based on HER2 status and sample type

HER2 status	Sample type	Total	HER2^homogeneous^	HER2^heterogeneous^
IHC 3+	Biopsy specimen	33	20 (60.6%)	13 (39.4%)
	Resected specimen	10	6 (60.0%)	4 (40.0%)
IHC 2+/FISH +	Biopsy specimen	3	2 (66.7%)	1 (33.3%)
	Resected specimen	2	0 (0.0%)	2 (100.0%)

Within the 5 IHC 2+/FISH+ patients, 3 demonstrated HER2 amplification in part of the tumor cells (20%, 20% and 50% respectively). These 3 were defined as HER2 heterogeneous.

Characteristics of biopsy specimens were also evaluated. The distribution of patients according to the numbers of biopsy specimens, tumor-containing fragments, and IHC 3+ fragments were shown in Table 3.

**Table 3:** Distribution of patients based on the numbers of biopsy specimens, tumor-containing fragments, and HER2 3+ fragments

	HER2^homogeneous^ (*n*)	HER2^heterogeneous^ (*n*)
Number of biopsy specimens		
4	5	2
5	3	2
6	5	4
7	3	0
8	2	1
9	1	1
10	1	1
12	1	1
13	1	0
15	0	1
16	0	1
Median (range)	6 (3–13)	6 (4–16)
Number of tumor-containing fragments		
2	0	1
3	1	1
4	8	4
5	4	3
6	4	1
7	1	1
8	2	0
9	0	1
12	2	1
14	0	1
Median (range)	5 (3–12)	5 (2–14)
Number of HER2 3+ fragments		
1	0	1
2	0	3
3	1	4
4	8	2
5	3	0
6	3	3
7	1	0
8	2	0
12	2	0
Median (range)	5 (3–12)	3 (1–6)

Abbreviations: ‘*n*’ refers to ‘patient number’.

### Efficacy and survival

The RR of the HER2^homogeneous^ group and the HER2^heterogeneous^ group was 46.4% (1 CR, 12 PR, 10 stable disease (SD), 5 progressive disease (PD)) and 55.0% (11 PR, 7 SD, 2 PD), respectively. No statistical difference was found between the two groups (*P* = 0.558).

**Figure 1:**
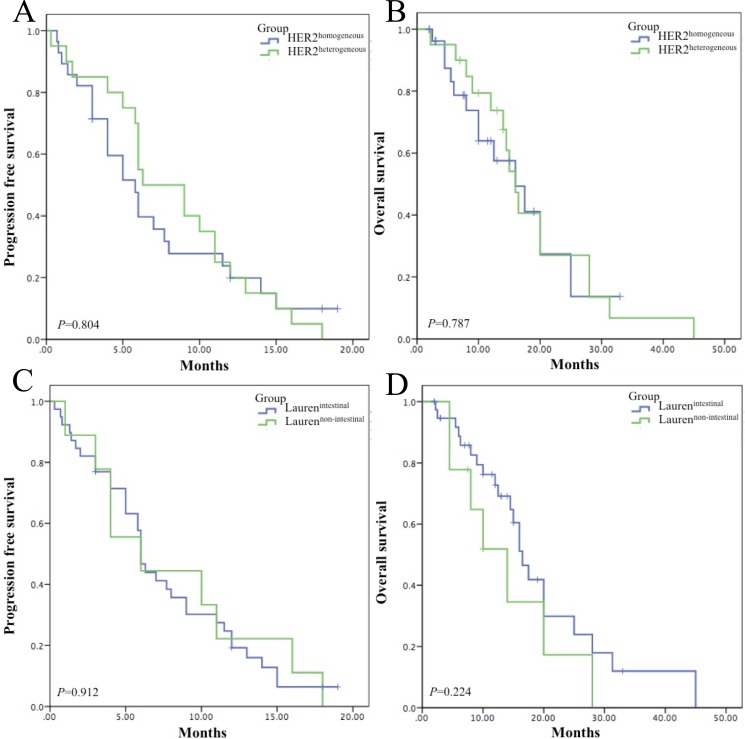
Survival analyses of HER2 heterogeneity and Lauren classification (**A**) Median PFS of the HER2^homogeneous^ group and the HER2^heterogeneous^ group was 5.80 months (95% CI 3.87–7.73) and 6.30 months (95% CI 1.92–10.68) without significant difference (*P* = 0.804). (**B**) Median OS of the HER2^homogeneous^ group and the HER2^heterogeneous^ group two groups were 16.00 months (95% CI 8.74–23.26) and 16.0 months (95% CI 13.59–18.41) with no statistical difference (*P* = 0.787). (**C**) Median PFS of the Lauren^intestinal^ group and the Lauren^non-intestinal^ group was 6.00 months (95% CI 5.42–6.59) and 6.00 months (95% CI 0.16–11.84) without significant difference (*P* = 0.912). (**D**) Median OS of of the Lauren^intestinal^ group and the Lauren^non-intestinal^ group was 16.50 months (95% CI 13.87–19.13) and 14.00 (95% CI 6.87–21.13) with no statistical difference (*P* = 0.224).

Median PFS demonstrated no significant difference between the HER2^homogeneous^ group and the HER2^heterogeneous^ group (5.80 months (95% CI 3.87–7.73) *vs* 6.30 months (95% CI 1.92–10.68), *P* = 0.804) (Figure 1A). As to OS, no statistical differences were reached either (16.00 months (95% CI 8.74–23.26) *vs* 16.00 months (95% CI 13.59–18.41), *P* = 0.787) (Figure 1B).

Both PFS and OS exhibited no statistical differences between Lauren subtypes. Median PFS in the Lauren^intestinal^ group and the Lauren^non-intestinal^ group was 6.00 months (95%CI 5.42–6.59) and 6.00 months (95% CI 0.16–11.84) (*P* = 0.912) (Figure 1C). Median OS of the two groups was 16.50 months (95% CI 13.87–19.13) and 14.00 (95% CI 6.87–21.13) respectively (*P* = 0.224) (Figure 1D).

Other factors including gender, age, differentiation, tumor location, number of metastatic sites and radical resection were also evaluated. None of them demonstrated statistical significance to affect PFS and OS.

### Survival analysis in subgroups

Survival analyses were further performed among subgroups divided based on combining HER2 heterogeneity and clinicopathological factors including gender, age, Lauren classification, differentiation, tumor location, number of metastatic sites and radical resection. Only HER2 heterogeneity combined with Lauren classification was associated with PFS and OS.

**Figure 2:**
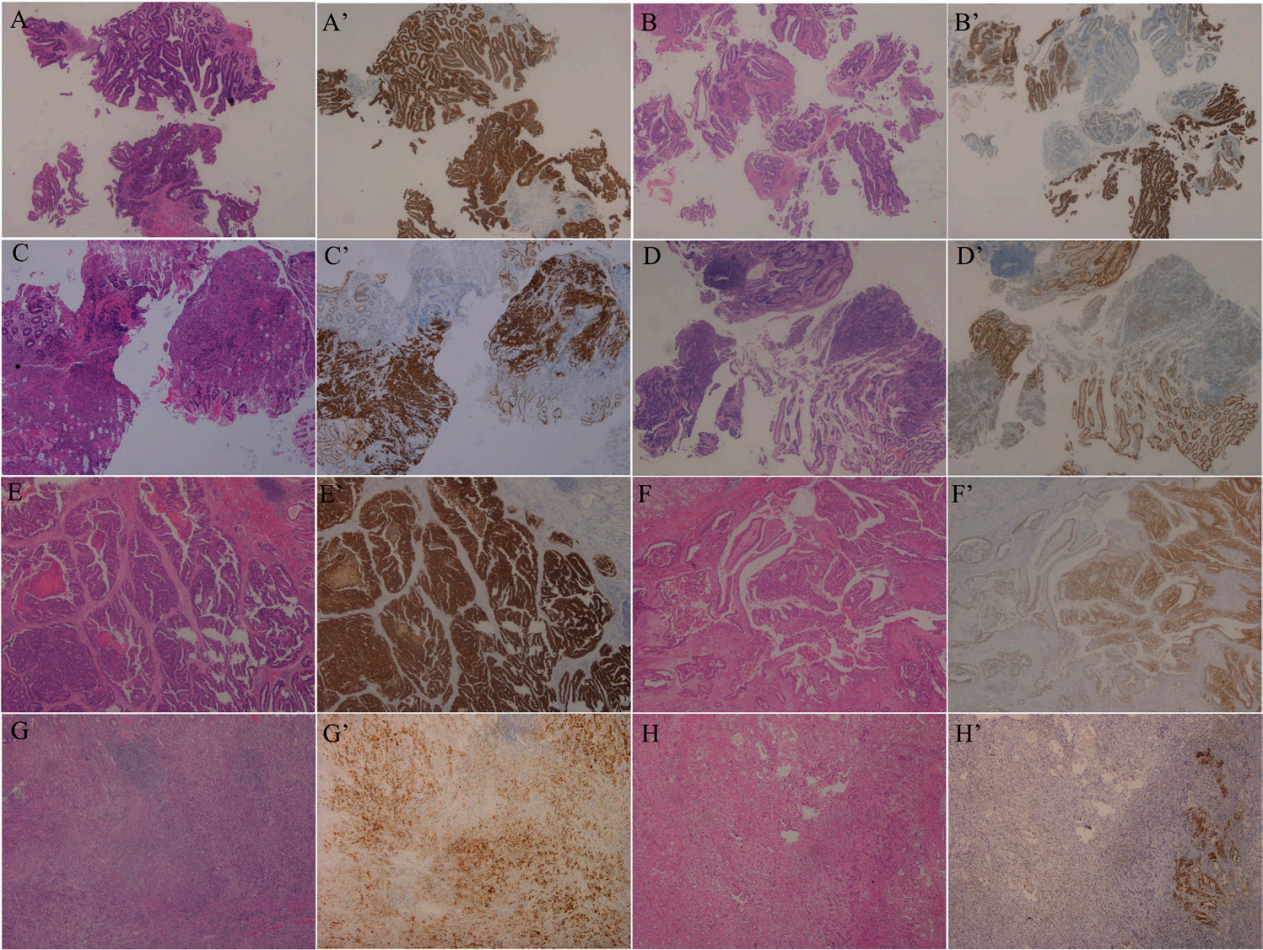
Examples of the 4 subgroups divided by combining HER2 heterogeneity and Lauren classification (HE and IHC, ×50) (**A**–**D**) Biopsy specimens. A, A’. HER2^homogeneous^/Lauren^intestinal^. B, B’. HER2^heterogeneous^/Lauren^intestinal^. C, C’. HER2^homogeneous^/Lauren^non-intestinal^. D, D’. HER2^heterogeneous^/Lauren^non-intestinal^. (**E**–**H**) Resected specimens. E, E’. HER2^homogeneous^/Lauren^intestinal^. F, F’. HER2^heterogeneous^/Lauren^intestinal^. G, G’. HER2^homogeneous^/Lauren^non-intestinal^. H, H’. HER2^heterogeneous^/Lauren^non-intestinal^.

**Figure 3:**
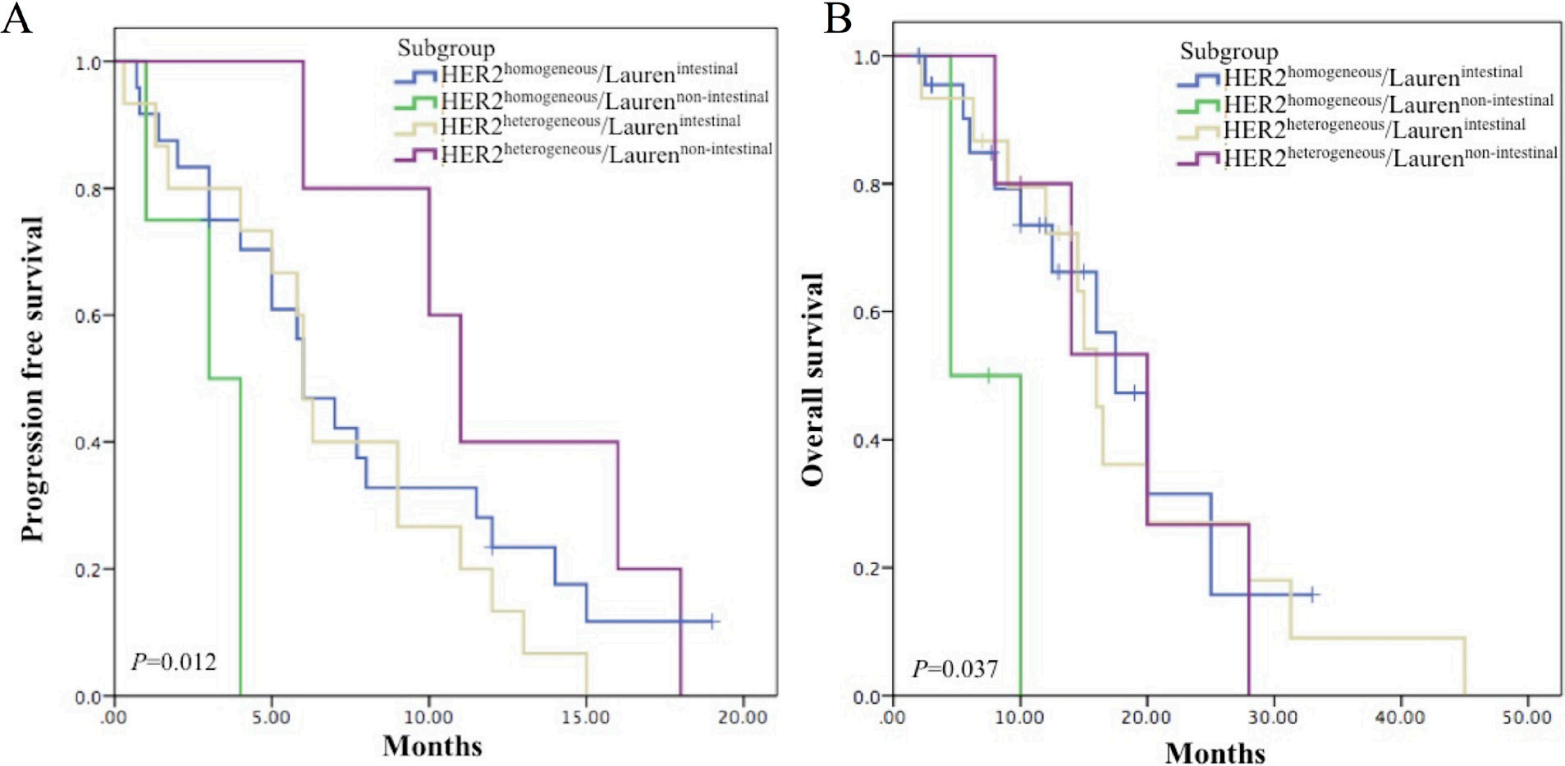
Survival analyses in subgroups by combining HER2 heterogeneity and Lauren classification (**A**) Kaplan–Meier curve for median PFS. Median PFS were 3.00 months (95% CI 1.04–4.96) in HER2^homogeneous^/Lauren^non-intestinal^ subgroup, 6.00 months (95% CI 4.22–7.78) in HER2^homogeneous^/Lauren^intestinal^ subgroup, 6.00 months (95% CI 5.37–6.63) in HER2^heterogeneous^/Lauren^intestinal^ subgroup and 11.00 months (95% CI 8.85–13.15) in HER2^heterogeneous^/Lauren^non-intestinal^ subgroup. The HER2^homogeneous^/Lauren^non-intestinal^ subgroup demonstrated the shortest PFS (*P* = 0.012). (**B**) Kaplan–Meier curve for median OS. Median OS were 4.5 months in HER2^homogeneous^/Lauren^non-intestinal^ subgroup, 17.50 months (95% CI 13.15–21.85) in HER2^homogeneous^/Lauren^intestinal^ subgroup, 16.00 months (95% CI 13.91–18.09) in HER2^heterogeneous^/Lauren^intestinal^ subgroup and 20.00 months (95% CI 10.04–29.96) in HER2^heterogeneous^/Lauren^non-intestinal^ subgroup. The HER2^homogeneous^/Lauren^non-intestinal^ subgroup showed the shortest OS (*P* = 0.037).

Based on HER2 heterogeneity and Lauren classification, the patients were divided into 4 subgroups: HER2^homogeneous^/Lauren^intestinal^, HER2^heterogeneous^/Lauren^intestinal^, HER2^homogeneous^/Lauren^non-intestinal^, and HER2^heterogenous^/Lauren^non-intestinal^ (Figure 2). PFS of the HER2^homogeneous^/Lauren^non-intestinal^ subgroup (3.00 months, 95% CI 1.04–4.96) was the worst among the 4 subgroups (*P* = 0.012) (Figure 3A), which was significantly shorter than that of the other patients together (6.30 months, 95% CI 4.81–7.79) (*P* = 0.003). In paired comparisons, PFS of the subgroup was shorter than HER2^homogeneous^/Lauren^intestinal^ subgroup (6.00 months, 95%CI 4.22–7.78)(*P* = 0.017), HER2^heterogeneous^/Lauren^intestinal^ subgroup (6.00 months, 95% CI 5.37–6.63) (*P* = 0.014) and HER2^heterogeneous^/Lauren^non-intestinal^ subgroup (11.00 months, 95% CI 8.85–13.15) (*P* = 0.004). The latter 3 subgroups showed no significant differences (*P* = 0.318).

Similar to PFS, OS of the HER2^homogeneous^/Lauren^non-intestinal^ subgroup (4.5months) was the shortest (*P* = 0.037) (Figure 3B), which was significantly shorter than that of the other patients together (16.50 months, 95% CI 13.64–19.36) (*P* = 0.004). Paired comparisons indicated that OS of the subgroup was shorter than HER2^homogeneous^/Lauren^intestinal^ subgroup (17.50 months, 95% CI 13.15–21.85) (*P* = 0.018), HER2^heterogeneous^/Lauren^intestinal^ subgroup (16.00 months, 95% CI 13.91–18.09) (*P* = 0.017) and HER2^heterogeneous^/Lauren^non-intestinal^ subgroup (20.00 months, 95% CI 10.04–29.96) (*P* = 0.041). No significant differences were reached among the latter 3 subgroups (*P* = 0.97).

### Evaluation of prognostic factors

Univariate analyses demonstrated that HER2 heterogeneity combined with Lauren classification was an independent prognostic factor in PFS (*P* = 0.031). HER2^homogeneous^/Lauren^non-intestinal^ tumor showed higher risk of progression than the other 3 subgroups, including HER2^homogeneous^/Lauren^intestinal^ subgroup (HR 3.887, 95% CI 1.135–13.309, *P* = 0.031), HER2^heterogeneous^/Lauren^intestinal^ subgroup (HR 4.678, 95%CI 1.161–18.845, *P* = 0.030) and HER2^heterogeneous^/Lauren^non-intestinal^ subgroup (HR 161.155, 95% CI 0.021–1254123.78, *P* = 0.266). The later 3 categories did not show significant difference (*P* = 0.349).

HER2 heterogeneity plus Lauren classification was also a prognostic factor in OS (*P* = 0.039). HER2^homogeneous^/Lauren^non-intestinal^ subgroup exhibited elevated risk of death than HER2^homogeneous^/Lauren^intestinal^ subgroup (HR 4.909, 95% CI 1.129–21.341, *P* = 0.034), HER2^heterogeneous^/Lauren^intestinal^ subgroup (HR 5.888, 95%CI 1.124–30.833, *P* = 0.036) and HER2^heterogeneous^/Lauren^non-intestinal^ subgroup (HR 7.866, 95% CI 0.753–82.182, *P* = 0.085). The latter 3 subgroups demonstrated no obvious difference (*P* = 0.972).

HER2 heterogeneity combined with other clinicopathological factors were also explored, including gender, age, tumor location, differentiation, number of metastatic organ, and radical resection. None of them were independent prognostic factors (Tables 4, 5). Two marginal values (*P* = 0.050) were identified in age and radical resection (Tables 4, 5), therefore, the two factors were subjected to multivariate analysis with Lauren classification. The results showed that only HER2 heterogeneity combined with Lauren classification was independent prognostic factor in PFS (*P* = 0.002) and OS (*P* = 0.013).

**Table 4:** Hazard ratios and 95% CI for the PFS and OS assessed by HER2 heterogeneity in different clinicopatholgical factors

	PFS		OS	
	HR (95% CI)	*P* value	HR (95% CI)	*P* value
Gender				
Male	1.165 (0.581–2.339)	0.667	1.193 (0.466–3.056)	0.712
Female	0.481 (0.122–1.892)	0.295	0.284 (0.034–2.397)	0.247
Age				
< 60	0.316 (0.100–0.999)	0.050	0.206 (0.021–1.999)	0.173
≥ 60	1.335 (0.631–2.824)	0.449	1.104 (0.445–2.740)	0.831
Tumor location				
GEJ	1.375 (0.469–4.028)	0.561	1.042 (0.272–3.995)	0.952
Other stomach	0.655 (0.311–1.380)	0.266	0.524 (0.190–1.443)	0.211
Lauren				
Intestinal	1.280 (0.647–2.534)	0.478	1.125 (0.472–2.685)	0.790
Non-intestinal	0.006 (0.000–48.289)	0.266	0.127 (0.012–1.328)	0.085
Differentiation				
Moderate	1.157 (0.550–2.433)	0.700	0.99 (0.385–2.532)	0.979
Poorly	0.486 (0.159–1.486)	0.206	0.417 (0.102–1.712)	0.225
Radical resection				
Yes	1.044 (0.520–2.094)	0.762	0.530 (0.074–3.831)	0.530
No	0.820 (0.226–2.975)	0.904	1.069 (0.461–2.481)	0.876
Number of metastasis				
< 3	1.070 (0.432–2.651)	0.884	0.877 (0.426–1.805)	0.722
≥ 3	0.758 (0.180–3.196)	0.706	0.982 (0.318–3.030)	0.975

Abbreviations: PFS: progression free survival; OS: overall survival; HR: hazard ratio; GEJ: gastric esophagus junction.

**Table 5:** Hazard ratios and 95% CI for the PFS and OS assessed by clinicopatholgical factors in different HER2 status

	PFS		OS	
	HR (95% CI)	*P* value	HR (95% CI)	*P* value
Gender(male *vs* female)				
HER2^homogeneous^	1.775 (0.746–4.222)	0.195	2.725 (0.826–8.989)	0.100
HER2^heterogeneous^	0.434 (0.098–1.921)	0.271	0.800 (0.219–2.921)	0.735
Age(< 60 *vs* ≥ 60)				
HER2^homogeneous^	0.499 (0.202–1.228)	0.130	0.53 (0.129–2.185)	0.380
HER2^heterogeneous^	1.39 (0.542–3.565)	0.493	1.009 (0.347–2.938)	0.987
Tumor location (GEJ *vs* other stomach)				
HER2^homogeneous^	1.918 (0.795–4.629)	0.147	3.219 (0.838–12.371)	0.089
HER2^heterogeneous^	0.838 (0.314–2.234)	0.723	1.306 (0.404–4.214)	0.656
Lauren (intestinal *vs* non-intestinal)				
HER2^homogeneous^	3.887 (1.135–13.309)	0.031	4.909 (1.129–21.341)	0.034
HER2^heterogeneous^	0.309 (0.088–1.087)	0.067	1.137 (0.355–3.642)	0.829
Differentiation (moderate *vs* poorly)				
HER2^homogeneous^	1.799 (0.692–4.679)	0.228	2.987 (0.863–10.362)	0.084
HER2^heterogeneous^	0.580 (0.216–1.559)	0.280	1.562 (0.537–4.542)	0.413
Radical resection (yes *vs* no)				
HER2^homogeneous^	0.689 (0.233–2.035)	0.500	0.670 (0.144–3.114)	0.610
HER2^heterogeneous^	0.32 (0.102–1.002)	0.050	0.47 (0.147–1.490)	0.199
Number of metastatis (< 3 *vs* ≥ 3)				
HER2^homogeneous^	1.015 (0.428–2.406)	0.973	1.345 (0.425–4.257)	0.614
HER2^heterogeneous^	1.332 (0.466–3.809)	0.593	0.627 (0.174–2.254)	0.474

Abbreviations: PFS: progression free survival; OS: overall survival; HR: hazard ratio; GEJ: gastric esophagus junction.

## DISCUSSION

Our study demonstrated that HER2^homogeneous^ group and HER2^heterogeneous^ group showed no significant discrepancies in PFS and OS, indicating that HER2 heterogeneity alone may not correlate with trastuzumab efficacy directly.

This finding provided indirect evidence that GCs with focal positive HER2 may also benefit from trastuzumab treatment. Currently, HER2 IHC 3+ was defined as strong reactivity in ≥ 10% of tumor cells. The 10% cutoff was not validated for GC, but rather adopted from HER2 scoring system for breast cancer. For cases with 3+ staining in < 10% of tumor cells, it is controversial. Some researchers figured out that the cutoff may lead to significant intra-observer variability [17]. Some recommended such cases should be subjected to *in situ* hybridization (ISH) test, and if amplification was detected, the case should be considered positive [6, 18]. A recent consensus from the College of American Pathologists, American Society for Clinical Pathology, and American Society of Clinical Oncology indicated that following the Ruschoff/Hofmann method, IHC results should be scored based on the staining pattern which accounted for ≥ 10% of tumor cells, and whether to perform ISH was determined by the IHC score [9]. Based on the current study, it might be reasonable to deduce that HER2 focal positive(< 10%) cases may also be eligible for trastuzumab treatment. Clinical trials are expected to explore this hypothesis.

The finding may potentially help expanding applicable people of trastuzumab treatment. It is of practical significance for GC, because HER2 positivity was only reported to range from 3.7% to 21.2% [10, 19, 20] and to be around 12–13% in Chinese population [11, 12]. Additionally, trastuzumab is one of the only two targeted regimens recommended by NCCN in GC currently [21], while most clinical trials aiming to find new targets failed to give positive results [21–23].

In our study, although both HER2 heterogeneity and Lauren classification alone may not be predictors for trastuzumab efficacy, subgroup analyses showed that by combining the two factors, the efficacy may be predicted. The HER2^homogeneous^/Lauren^non-intestinal^ subgroup demonstrated the worst prognosis.

Both Lauren classification and HER2 status are important clinicopathological factors with prognostic values in GC. The intestinal type GCs demonstrate better prognosis [24]. With regard to HER2, its validity as a prognostic factor is still controversial [23, 25]. According to a systematic data analysis of literature, most studies favored HER2 as a negative prognostic factor [26]. However, the prognostic values and predictability to trastuzumab response of HER2 heterogeneity were not studied in GC before. Studies of HER2 genetic heterogeneity in breast cancer generated inconsistent findings. Lee et al. found out that the heterogeneity was a negative predictor of trastuzumab response [13, 27]. However, Gullo et al. reported findings to the contrary [28]. Furthermore, a predictive relationship between the genetic heterogeneity and trastuzumab response was not found in early stage breast cancers in the adjuvant setting [29]. Collectively, these findings indicated that the impacts of HER2 heterogeneity on trastuzumab efficacy are not fully understood and warrant further studies. Researches of GC cell lines indicated that HER2 up-regulation increased cell migration, invasion and metastasis [30]. Therefore, compared with HER2^heterogeneous^ counterpart, more tumor cells in HER2^homogeneous^ positive GCs showed more aggressive biological behavior. The co-existence of the two adverse factors including HER2^homogeneous^ and Lauren^non-intestinal^ may be a possible explanation of the poor response to trastuzumab treatment.

To our knowledge, this is the first study exploring the impact of HER2 heterogeneity and its combination with Lauren classification on treatment outcomes of trastuzumab in GC. Combining Lauren classification and HER2 status as a prognostic factor has been studied in GCs without trastuzumab treatment. He et al. found that HER2 was a prognostic factor in intestinal type GCs but not in diffuse/mixed subtype GCs [31]. Qiu et al. figured out that Lauren classification combined with HER2 status was a better prognostic factor, and intestinal type GCs with HER2 negative had the best survival, while diffuse type with HER2 positive had the worst [24]. These previous studies, including ours, indicated that when discussing the prognostic value of HER2, Lauren classification should be considered.

In this study, HER2 status of 36 patients was evaluated in biopsy specimens which have been shown to be highly consistent with surgical specimens [32–34]. To predict HER2 precisely, 4 tumor-containing fragments were recommended [35]. In our study, 33 of the 36 patients had ≥ 4 tumor fragments. Therefore, we believe biopsy specimens in the study can reflect the actual HER2 status.

Our study has several limitations. First, its retrospective nature from a single institution. Second, sample size is relatively small (48 patients), especially in the Lauren^non-intestinal^/HER2^homogeneous^ subgroup (4 patients). This is partly due to that HER2 positive GCs are mainly found in intestinal type tumors. The small sample size indicates that findings of the subgroup and related clinical significance warrant further observation. Third, little was known about the predictability of biopsy specimens in assessing Lauren type currently.

In conclusion, the main finding of the study is that HER2 heterogeneity alone may not correlate with the efficacy of trastuzumab therapy directly in HER2 positive advanced GCs. Additionally, using two simple clinicopathological factors by combining HER2 heterogeneity and Lauren classification may help to identify a subgroup with poor response to trastuzumab treatment which is homogeneous HER2 positive and non-intestinal type.

## MATERIALS AND METHODS

### Ethics statements

The research protocols were approved by the ethics board of Zhongshan Hospital. Prior written informed consents were collected from all patients.

### Patients and treatment

Patients with HER2 positive advanced GC who accepted trastuzumab treatment were retrospectively collected from February 2010 to March 2016 in Zhongshan Hospital, Fudan University. Both inoperable patients during their first visit and recurrent/metastatic patients after radical resection were included. All the patients received standard palliative treatment including trastuzumab with a dose of 6 mg/kg every 3 weeks after a first infusion of 8 mg/kg. There were no protocol-specified chemotherapy regimens.

Patient characteristics including gender, age (< 60 or ≥ 60), tumor location (gastric esophagus junction (GEJ) or other stomach), Lauren classification (intestinal or non-intestinal), differentiation, number of metastatic organs (< 3 or ≥ 3), and radical resection (yes or no) were collected.

### Pathological evaluation, immunohistochemistry (IHC) staining and fluorescence *in situ* hybridization (FISH)

All cases were reviewed by two experienced gastrointestinal pathologists. For inoperable patients, biopsy specimens were used to evaluate HER2 heterogeneity. All the biopsy specimens were subjected to the analysis if multiple biopsies were performed. For patients with resected specimens, to determine HER2 heterogeneity, all the tumor-containing paraffin blocks were subjected to HER2 assessment.

HER2 staining was performed with an iView DAB Detection Kit (Ventana, Tucson, AZ) on a BenchMark XT automated stainer (Ventana Medical Systems, Inc.,Tucson, AZ) according to the procedures previously described [36]. HER2 (4B5) rabbit monoclonal antibody (Ventana Medical Systems, Inc., Tucson, AZ) was used.

For HER2 IHC 2+ patients, FISH was performed on a selected section with the strongest HER2 intensity for each case. A Pathvysion HER2 DNA Probe Kit (Abbott Laboratories, Des Plaines, Illinois) was used according to the manufacture`s instructions.

### HER2 assessment

HER2 status was assessed by 2 independent observers, and was verified by a discussion panel consisting of 3 observers on occasion of discrepancies. All observers were blinded with regard to aim of the study. HER2 positive was defined as IHC 3+ or IHC 2+ plus FISH positive (IHC 2+/FISH+).

HER2 was scored according to the established IHC criteria for GC [6]. Briefly, for biopsy specimens, 0 was defined as no membranous reactivity in any tumor cell; 1+ was defined as tumor cell cluster (≥ 5 cells) with a faint membranous reactivity; 2+ was defined as tumor cell cluster (≥ 5 cells) with a weak to moderate complete, basolateral, or lateral membranous reactivity; 3+ was defined as tumor cell cluster (≥ 5 cells) with a strong complete, basolateral, or lateral membranous reactivity. For resected specimens, 0 was defined as no staining or < 10% tumor cell positive staining; 1+ was defined as faintly staining on ≥ 10% tumor cell membrane and in part of their membrane; 2+ was defined as weak to moderate complete, basolateral, or lateral membranous reactivity in ≥ 10% of tumor cells; 3+ was defined as strong complete, basolateral, or lateral membranous reactivity in ≥ 10% of tumor cells.

In FISH assessment, 5 areas were analyzed in each case. 20 cancer cell nuclei were evaluated in each area. HER2 amplification was considered positive when a ratio of HER2: CEP17 (centromeric probe 17) of ≥ 2, or when HER2 signal clusters were observed.

HER2 homogeneity and heterogeneity was defined as follows. For IHC assessment, HER2 homogeneous expression was defined as uniformly 3+ staining in all tumor cells in biopsy specimens and in over 90% of tumor cells in each block in resected specimens. Cases that did not meet the criteria were considered HER2 heterogeneous. For FISH assessment, the definition of genetic heterogeneity of breast cancer was adopted [37]. The heterogeneity was defined as the presence of tumor cells with a HER2/CEP17 ratio ≥ 2 in 5% to 50% tumor cells. If the more than 50% tumor cells had a ratio of ≥ 2, the tumor was considered homogeneous.

### Follow-up

Follow-up data was acquired from medical records. CT/MRI was performed for response evaluation every 8 weeks based on Response Evaluation Criteria in Solid Tumors (RECIST)(version1.1) or earlier when indications of treatment failure were present. Patients were considered on study until death or loss to follow-up. The last date of follow up was March 16th, 2016.

### Statistical analysis

Progression free survival (PFS) was defined as the time from the start of transtuzumab administration to the date of tumor progression. Overall survival (OS) was defined as the time from the start of transtuzumab treatment to death. Response rate (RR) was defined as complete response (CR) plus partial response (PR).

Kaplan–Meier curves were constructed for survival analysis. Clinicopathological characteristics were calculated with Chi-squared tests, Fisher's exact tests, and Mann-Whitney *U* test. Variables associated with prognostic value were selected to evaluate by univariate and multivariate analysis using Cox proportional hazard regression models. Data were presented as an HR and a 95% CI. Stratified log-rank tests and Cox regression analyses were also carried out in subgroup analyses.

For each analysis, a two-sided *P*-value of < 0.05 was considered statistically significant. Analyses were conducted using SPSS software (version 19.0; SPSS, Chicago, IL, USA).

## Abbreviations

GC: gastric cancer
GEJ: gastric esophagus junction
IHC: immunohistochemistry
FISH: fluorescence *in situ* hybridization
RR: Response rate
PFS: progression free survival
OS: overall survival
SD: stable disease
PD: progressive disease
CR: complete response
PR: partial response.
